# A rapid review of evaluated interventions to inform the development of a resource to support the resilience of care home nurses

**DOI:** 10.1186/s12877-023-03860-y

**Published:** 2023-05-05

**Authors:** Anita Mallon, Gary Mitchell, Gillian Carter, Derek McLaughlin, Christine Brown Wilson

**Affiliations:** School of Nursing and Midwifery Queens University Belfast, University Rd, Belfast, BT7 1NN Northern Ireland

**Keywords:** Resilience, Nursing, Intervention, Evaluation, Care homes, Care home nurses

## Abstract

**Backgound:**

Nurses working in care homes face significant challenges that are unique to that context. The importance of effective resilience building interventions as a strategy to enable recovery and growth in these times of uncertainty have been advocated. The aim of this rapid review was to inform the development of a resource to support the resilience of care home nurses. We explored existing empirical evidence as to the efficacy of resilience building interventions. undertaken with nurses.

**Methods:**

We undertook a rapid review using quantitative studies published in peer reviewed journals that reported resilience scores using a valid and reliable scale before and after an intervention aimed at supporting nurse resilience. The databases; Cumulative Index to Nursing and Allied Health Literature, Medline and PsychInfo. and the Cochrane Library were searched. The searches were restricted to studies published between January 2011 and October 2021 in the English language. Only studies that reported using a validated tool to measure resilience before and after the interventions were included.

**Results:**

Fifteen studies were included in this rapid review with over half of the studies taking place in the USA. No studies reported on an intervention to support resilience with care home nurses. The interventions focused primarily on hospital-based nurses in general and specialist contexts. The interventions varied in duration content and mode of delivery, with interventions incorporating mindfulness techniques, cognitive reframing and holistic approaches to building and sustaining resilience. Thirteen of the fifteen studies selected demonstrated an increase in resilience scores as measured by validated and reliable scales. Those studies incorporating ‘on the job,’ easily accessible practices that promote self-awareness and increase sense of control reported significant differences in pre and post intervention resilience scores.

**Conclusion:**

Nurses continue to face significant challenges, their capacity to face these challenges can be nurtured through interventions focused on strengthening individual resources. The content, duration, and mode of delivery of interventions to support resilience should be tailored through co-design processes to ensure they are both meaningful and responsive to differing contexts and populations.

**Supplementary Information:**

The online version contains supplementary material available at 10.1186/s12877-023-03860-y.

## Introduction

The job of nursing is complex and the health and social care environment increasingly challenging [1, 2]. The COVID 19 pandemic has brought these challenges to a different level of complexity resulting in a high psychological burden on nurses [3–5]. A job resource and protective factor that can help mediate some of the stresses of these work-based challenges is resilience [6, 7].

Resilience focuses on the positive aspects of individuals to act as a buffer through adversity, to not only allow recovery but enable people to positively manage challenges, learning and adapting from the experience [8, 9]. Resilience may be considered as the ability to adapt to adversity [10] as a dynamic process [11] and an innate resource [12]. The results of a systematic review of resilience interventions concluded that resilience could be conceptualised as mental health in relation to ‘stressor load’ [13]. Our understanding of resilience is informed by a broad systems approach that incorporates the biopsychosocial model of resilience [14] with a socio-ecological perspective [15]. It refers to not only individual capabilities but systems that can ‘neutralise’ adversities and its affects [14] While many definitions of resilience exist, core characteristics of resilience are: the presence of adversity as an antecedent and positive adaptation as a consequence, this differentiates resilience from other more common concepts such as coping and hardiness [16]. Resilience may be seen to differ from coping as it influences the appraisal of a situation before employing specific strategies to cope with the situation [16]. It differs from common understandings of hardiness as when people use the term ‘hardy’ they refer to an ability to withstand stressors but the positive adaptation may or may not be present. As such these concepts relate to resilience but alone do not address its core characteristics. Attributes and assets within and around the person can influence and facilitate this adaptation, this assets-based approach distinguishes resilience interventions from those that look at stress reduction and management of burnout [17].

While resilience is variously interpreted within different populations and contexts [18] a concept analysis of resilience as it applies to nursing defines resilience as the ability to adapt, avoid psychological harm while providing optimum care [19]. Studies highlight that nurse resilience can protect against psychological harm and higher resilience can predict increased levels of happiness, wellbeing and may increase job satisfaction and retention [20, 21]. Reviews of empirical data suggest that resilience can act as a buffer against burnout [22], exhaustion, anxiety, and depression [23]. Interventions aimed at increasing resilience among nurses have proliferated in recent years with resilience considered as core to professional practice [24] and an essential graduate capability [25]. In one of only two studies focusing on care home nurses included in a recent review of nurse resilience [26] resilience was associated with higher perceived quality of care [27]. However, variability in the meaning of resilience and therefore what exactly is being measured prohibits the building of a framework that may help to understand exactly what resilience means as a nurse and what strategies may help build resilience.

Reviews measuring the effectiveness of resilience based interventions have tended to focus on health care workers in general with each recommending that interventions be tailored to specific context and populations [7, 17, 28, 29]. Only two reviews were found relating to the effectiveness of interventions specific to nursing [30, 31]. A mixture of mindfulness, yoga, cognitive reframing exercises, and problem solving exercises formed the basis of resilience interventions. Teaching on how to improve knowledge and response to stress were also significant factors [30, 31]. A meta-analysis of resilience training interventions aiming to influence nurse resilience demonstrated that resilience training increased resilience scores but variations in assessment tools and a variety of outcome measures negated any generalisation of effect [31]. Kunzler et al. extracted studies focusing on nurses from a Cochrane review of psychological intervention to build resilience in health care professionals [17, 30]. They found some evidence of positive short term effects of resilience training and interventions on nurse resilience and mental health outcomes. However as with other reviews in the broader health care literature, the heterogeneity of measurement tools and outcomes assessed made it difficult to decide what elements of an intervention are most effective [7, 17, 30–32]. Despite the advocacy for targeted and context specific interventions [7, 13, 17, 33], and the recognition that resilience is a core protective factor against psychological harm [20, 34, 35]. A preliminary scope of the literature revealed no evaluated interventions aimed at supporting nurse resilience specific to the context of the care home. The aim of this rapid review was to inform the development of a resource to support the resilience of care home nurses by extracting empirical evidence as to the efficacy of resilience building interventions undertaken with other nurse populations.

## Methodology

The World Health Organisation highlight the importance of timely reviews using rigorous search and transparent reporting measures to inform health policy detailing how and in what settings these programmes work [36]. In a recent overview of definitions, a rapid review is identified as;

"...*a form of knowledge synthesis that accelerates the process of conducting a traditional systematic review through streamlining or omitting a variety of methods to produce evidence in a resource-efficient manner.”* [37] p80.

A checklist was used in the design, planning, execution, and evaluation of this review to ensure ‘replicability and objectivity’ within a limited time scale [38, 39] (Additional file 2 Brief Review Checklist).

### Search strategy

The search was an iterative process with the refinement of the review aims throughout the search process. Measuring resilience as a primary outcome using a valid and reliable tool was the recommendation from reviews looking at resilience across populations. Therefore, in this rapid review a systematic search extracted studies that used a measure of resilience that had undergone psychometric testing to determine reliability and validity as reported in the selected papers. As studies relating to resilience interventions in nurses have emerged primarily in the past 10 years [1] and to understand the current state of evidence, studies published between January 2011 and October 2021 were retrieved from the following sources: The Cumulative Index to Nursing and Allied Health Literature (CINAHL) sought to extract studies specific to the discipline of nursing. Medline gave a more general picture related to medicine and PsycInfo. gave some insight into the psychological literature therefore including the breadth of the evidence base of the psychological and social sciences. Reference lists of key papers were screened to identify additional studies that met the inclusion criteria (see Table 1).

**Table 1:** Inclusion/ exclusion criteria

Inclusion criteria	Exclusion criteria
Peer reviewed publications	Discussion papers, editorials, and reviews
Interventions developed specifically for registered nurses working in primary secondary and tertiary care aiming at supporting resilience	Studies where the participants were not registered nurses
Intervention studies using a quasi-experimental design, pre and post-test design, randomised control trials and trials with a control list.	Studies without an intervention
The use of a valid and reliable tool to measure resilience before and after an intervention aiming to affect resilience levels	Studies without a valid and reliable measurement tool
Articles published in the English language only	Articles published in a language other than English
Studies published from 2011 onwards	Studies published prior to 2011

**Fig. 1:**
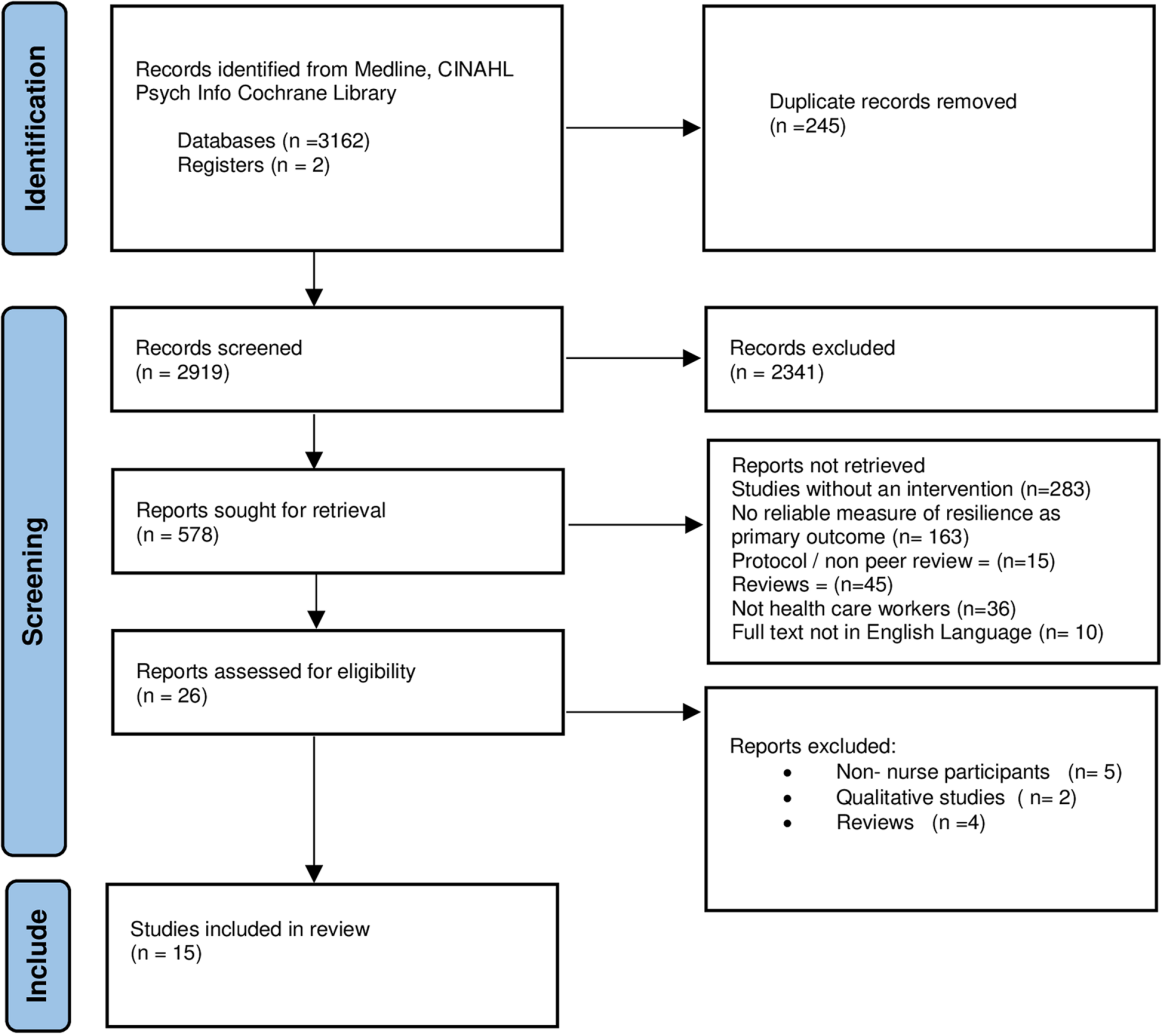
Adapted PRISMA [40] detailing the search and retrieval process

An experienced subject librarian was consulted regarding specific terms and resultant combinations using Boolean operators (OR/AND). The following MESH terms and combinations were used relevant to each database: a. Nurse, (MH) b. Resilience, c. Intervention, d. Evaluation. The systematic search of the databases was undertaken by one author (AM) between September and October 2021. This followed the guidance of the Preferred Reporting Items for Systematic reviews and meta-analyses (PRISMA) [40]. Initial searches tested the search terms and combinations to ensure retrieval of relevant studies on each database. A full description of the search history for the PsycInfo database is included (Additional file 1 Search PsychInfo). Relevant changes were made to key terms in keeping with the database requirements. The results from the database searches were imported into EndNote v 20 and duplicates were removed. Adapted PRISMA below (Fig. 1) details the search process.

Two authors (AM /CBW) independently reviewed title/abstract of extracted studies using the inclusion and exclusion criteria (Table 1). The researchers (AM CBW) met and critically appraised the remaining studies inviting support from a third researcher (GM) when differences were difficult to resolve. Reasons for final exclusion are presented in the PRISMA chart [40].

Key data from the final studies were independently extracted and key characteristics placed in a table by AM and CBW under the following headings: a. author/ year and country, b. study objectives, c. population and setting, d. design, e. intervention f. resilience scales and other outcome measures g. duration and h. main study findings. The characteristics of the included studies are presented in Table 2. A narrative synthesis of the data was undertaken.

**Table 2:** Key characteristics of included studies

Author/year / country	Objectives	Settings and participants	Study design	Intervention	Resilience measures	Duration	Main findings
Andersen, et al., 2021 [41] USA	1) to replicate the research based on the pilot study, 2) to increase resilience in nurses working on all units at four hospitals and. 3) to determine which interventions were preferred and most effective	4 urban hospitals 328 nurses enrolled with 167 completing T2	Cross sectional longitudinal quasi experimental survey Oct 2018-Jan 2019	Tool kit with aromatherapy stick. Adult colouring book and pencils a mind game puzzle book recommendations for deep breathing Individual Mind activity book gaming apps	Connor-Davidson Resilience Scale (CD-RISC) 10 **Other outcomes** Demographics and a stress scale (1–10)	10 shifts 4–6 weeks 3 time points baseline (T1) 4–6 weeks (T2) 3 months (T3)	Paired t test CD-RSIC significantly increased at T2 and T3 T2 (df 166 = t-4.87, *p* < .001) and further increased at T3 (df 71 = t-5.88, *p* < .001). Increases in CDRISC 10 scores from T2 to T3 were not significant. Aromatherapy pen used most often by younger and breathing by older participants
Babanataj et al. 2019 [42] Iran	To determine the effect of the training on occupational stress and resilience in nurses working in ICUs.	30 nurses in 7 critical care units in one teaching hospital	Quasi experimental intervention study April 2015-Feb 2016	Training programme of five sessions between 90 and 120-minute duration covering characteristics of resilient people, supportive factors and methods for improving resilience. Group training	CD-RISC (25) **Other outcomes** Demographics Expanded Nursing Stress Scale (ENSS)	Pre/post test	The mean score of resilience Increased significantly after the intervention (*P* = .001; T value − 13.74; df 29). Pre mean 67.9 post mean 81.4
Bernburg et al. 2019 [43] Germany	To explore effectiveness of stress management and coping skill training for mental health nurses.	85 mental health nurses working in psychiatric hospitals: 44 in the intervention group and 42 in waitlist control	RCT with waitlist control group	12 weeks training of 1.5–2 hours covered work-related stress management training, problem solving techniques, and solution-focused counseling. Mindfulness, solution focused CBT	Brief resilient coping scale **Other outcomes** Perceived Stress Questionnaire	Baseline T1 3 months T2 6 months T3 12 months	Resilience At T1 and T2, the majority of effect sizes were in the range of large to medium (T1: d.0.8; T2 d.0.5 T1 3 months 3.11 T2 6 months 3.01 T3 12 months 2.94 (sig effect size (T1: d-0.8; T2 d-0.5. No increase in longer term Significant positive changes in perceived stress, emotional regulation skills and self-efficacy. Scores on perceived quality of patient relations were higher after the intervention
Blackburn et al. 2020 [44] USA	To develop an evidence-based program for addressing the concerns of burnout and secondary trauma and building on the concept of resilience in Oncology healthcare providers.	164 oncology staff, of which 160 = nurses One oncology centre	Evidence based quality improvement programme	THRIVE© program, which consists of an eight-hour retreat designed to teach self-care strategies (diaphragmatic breathing; art for self-care; music for self-care; mindfulness; sleep; chair yoga; aromatherapy; guided imagery; acupressure and self-massage), a six-week private group study Interaction on a social media platform, and a 2 h wrap-up session.	CD-RISC (25) **Other measures** Compassion Fatigue Short Scale (burnout and secondary trauma)	Pre/ post test 2mths//4mths and 6 mths	CD-RISC scores increased significantly pre- to post program assessment (t = 2.64, df = 9, *p* = 0.0268). Pre 74 Post 85 2mths = 83 4 months = 82 6 months = 86 Other outcomes Burnout and secondary trauma scores decreased significantly from pre to post assessment
Chesak et al. 2015 [45] USA	To examine outcomes of a brief Stress Management and Resiliency Training (SMART) program within a nurse orientation program.	Nurses who were new to the institution or transitioning to a new unit or new role and who were undergoing nurse orientation in one centre. 55 recruited (27 intervention; 28 control) 40 completed study (19 intervention; 21 control)	RCT pilot study	SMART study comprises 90 minute session on mind-body approaches to stress management, developing intentional attention, practicing gratitude, compassion- followed by a 1 hour follow up sessions and bi-weekly handouts 12 week intervention	CD-RISC. (25) **Other outcomes** Perceived Stress Scale (PSS), the Mindful Attention Awareness Scale (MAAS), the Generalized Anxiety Disorder 7-item scale (GAD-7), and	Baseline and 12 weeks following intervention	.Pre intervention mean 79.68 Post intervention mean 79.7 (12 weeks) Mindfulness and resilience scores improved in the IG and declined in the CG (but not significantly). Changes in mindfulness, anxiety, and resilience did not differ significantly between the groups. Positive changes with potential for efficacy
Chesak et al. 2021 [46] USA	Aims: 1) assess the feasibility of integrating a Stress Management and Resiliency Training (SMART) program within a nine-month pilot nurse residency program and 2) assess the effects of the program on participants’ _stress, anxiety, mindfulness, and resilience relative to a comparison group.	Pilot nurse residency programme at one academic medical centre (< 1 yr experience) 51 nurses Intervention: *n* = 23; Comparison *n* = 28 Difference in demographics of the comparator group was that they could be in any unit including ICU Attrition not an issue as this was incorporated into a programme	Quasi experimental feasibility (convenience sample)	SMART study 90 mins session psychology and neurobiology of stress and resilience SMART principles; gratitude, compassion, acceptance meaning and purpose forgiveness, celebration and reflection facilitated by nurse specialist and nurse ed. Follow up monthly sessions for 7 moths with last one on reflection. Workbook: topics pertaining to the sessions Specialist.acceptance Intervention arm compensated at usual RN salary so not in their own time and provided with protected time	CD-RISC (10) item (0–40) Other measures Perceived stress scale Generalised anxiety disorder scale Mindful attention awareness scale	CDRISC 10 B 26.5 C 30.4 T1. 27.5 29.1 T2. 28.4 29.6 T3 29.9 30.8 T4 32.3 29.9 *P* < 0.001 p.95	Significant improvements were noted for resilience (*P <* .001) in the intervention group compared with the comparison group Resilience levels increased steadily in the intervention group but not in the comparator group. However resiliency was stronger to begin with in the comparator group The effect of time on resilience (measured by CD-RISC scores) was significantly different between the intervention and comparison groups (*P <* .001 for the time × _group interaction) plus a significant increasing linear effect in the intervention group (average increase in score between each time point was 1.42 [95% CI, 1.06 to 1.78; *P <* .001]) but saw no effect within the comparison group (*P* = .95) Support for the inclusion in a residency programme **Other outcomes** Improvements in stress and mindfulness (*p* < 0.001**)**
Craigie et al. 2016 [47] Australia	To evaluate the feasibility of a mindfulness-based intervention aimed at reducing compassion fatigue and improving emotional well-being in nurses.	21 nurses recruited from a large teaching hospital in Western Australia. 20 completed study	Pre/post with 1 month follow up	Mindful Self-care and resiliency (MSCR) intervention: 1-day compassion fatigue prevention educational workshop, followed by a series of weekly mindfulness training seminars conducted over 4 weeks (12 h total intervention time). Compassion fatigue resiliency	CD-RISC).10 item range 0–40 **Other measures** Patient Health Questionnarie9, PTSD, CAGE questionnaire, Quality of Life (ProQOL-5), DASS-21, Anxiety, Passion for Work	Pre/post I month	No significant changes were observed for general resilience, post-intervention or at follow-up. Mean 28.2, 29.6 and 28.7 **Other outcomes** Significant improvements in Compassion Satisfaction, Burnout, Trait negative effect, Obsessive compassion and Stress
Delaney 2018 [48] UK	To assess the benefits of Mindful Self-Compassion (MSC) training intervention on nurses	18 nurses in one hospital recruited and 13 completed the training	Observational mixed research pilot study using evaluation design framework	Eight week Mindful Self-Compassion (MSC) training intervention for 2.5 hours – emphasis on self-compassion, with a secondary emphasis on mindfulness. Participants received four practice CDs of formal practices and informal practices that they could use on the job.	CDRISC (25) **Other measures** Freiburg Mindfulness inventory ProQOL Version 5 Professional Quality of Life Scale: Compassion Satisfaction and Fatigue Version	Pre/post	Resilience results: pre mean 67.61 SD .8.79 post mean 80.30 SD 8.08 Effect size Cohen d 1.50; 95% CI [0.27–2.73] (large) **Other outcomes** Strong negative association between participants’ reported enhanced mindfulness and secondary traumatic stress and burnout. With an increase in mindfulness, there was a decrease in secondary traumatic stress and burnout. These reported results also show a large positive association between mindfulness and resilience *r* = 0.66. Enhanced mindfulness was a predictor of reduced secondary traumatic stress β = −.47 and also burnout β = −.42. Conversely the increased reported score for mindfulness was predictor of a large increase in enhanced resilience β = 1.09. Significant Increase in compassion satisfaction score
Foster et al. 2018 [49] Australia	The aim of this study was to evaluate the feasibility of a workplace resilience education programme (PAR) for nurses in high-acuity adult mental health settings.	Nurses working in 2 acute mental health inpatient units Purposive sample *n* = 24	A feasibility study Single-group pre-test post-test design with follow-up at 3 months post intervention.	Two days Face to face training delivered 3 weeks apart Evidence based and multimodal integrating cognitive behavioural and interpersonal therapy perspectives in a manualised format	WRI (Work place Resilience Inventory 60 items). **Other outcomes** Mental Health DASS 21; Wellbeing – satisfaction with life scale; Ryffs scale of psychological wellbeing; Satisfaction with work (1 item); Coping self-efficacy;	T1 baseline T2 after 1st full day workshop T3 3 months after 2nd workshop	Participants reported relatively strong workplace resilience at the commencement of the study. At T3 strong + correlation between Self regulatory: cognitive subscale of WRI and coping self-efficacy and satisfaction with work **Other outcomes** There were strong and very strong correlations among the Self-Regulatory: Behavioural subscale of the WRI and (i) Stress, (ii) Coping Self-Efficacy, and (iii) Satisfaction with Work.This indicated that when self-regulatory behaviours were low, stress was high (negative correlation; These findings indicated that PAR participants appeared better able to understand and control negative and ineffective behaviours and thoughts after the intervention..
Grabbe et al. 2020 [50] US	To test the effectiveness of a 3 hour Community Resiliency Model (CRM) training on the well-being, resiliency, secondary traumatic stress, burnout, and physical symptoms of nurses	Registered nurses in two tertiary care hospitals (*n* = 196) Allocated to CRM *n* = 99 Allocated to control (nutrition intervention) *n* = 97 40 intervention and 37 control completed study	Randomised control trial parallel design	CRM is a simple, Innovative, self-care program that focuses on somatic experience of stress. Mental well-being is enhanced through the use of sensory awareness skills – non cognitive variant of mindfulness. CRM teaches six skills to enhance attention control while tracking body sensations before and after using the techniques	Connor Davidson Resilience Scale-10 (CD-RISC), **Other outcomes** the WHO-5 Well-being Index (WHO-5), the the Secondary Traumatic Stress Scale (STSS), the Copenhagen Burnout Inventory (CBI), and the Somatic Symptom Scale-8 (SSS-8).	Baseline 1 week 3 months 1 year	The outcomes that significantly changed (and improved) over time were well-being (*p* = .006), resilience (*p* = .004), secondary traumatic stress (STSS) (*p* = .009), and somatic symptoms (SSS-8) (*p* = .004). (Moderate-to-large effect sizes) Posthoc tests were performed for time within each group. All of the improvements over time for these four outcomes were for the CRM intervention group (well-being (F(3, 211.220) = 4.993, *p* = .002), resilience (F(3, 193.8) = 2.689, *p* = .048), secondary traumatic stress (F(3, 204.0) = 2.504, p =.060), and somatic symptoms (F(3, 191.2) = 3.185, *p* = .025)), with no significant time effects for the control group (*p* > .10 for all post hoc time effect tests)
Im et al. 2016 [51] Korea	To evaluate effects of huddling programme on Empowerment organisational commitment and ego resilience	49 nurses with < 5 years nursing experience working in 2 general hospitals	Randomised control trial Control group (*n* = 25) Experimental (n-24)	Four sessions of huddling programme. over 9 weeks Mentorship Full day retreat and after work social network Jan and Feb 2013 Creative expression, team work, sharing, mentorship. Focus on group autonomy and cohesiveness. Online messages of support, mutual encouragement and inquiries about group members.	Ego resilience (Block and Kremen 1996) **Other measures** Demographics Empowerment (Spreitzer) Organisational commitment (Allen and Myer 1990	Pre and post after 4 weeks	No significant differences in the subcategories nor overall resilience scores Mean CG– 45.60 IG 45.71 *P* < 0.957 no indication of pre and post means for each group Groups were homogenous in terms of demographics and concept of resilience **Other outcomes** Normative Commitment and impact of empowerment higher in experimental group
Lin et al., 2019 [52] China	Evaluate the effects of a modified mindfulness-based stress reduction (MBSR) program on the levels of stress, affect, and resilience	110 nurses from two general hospitals Intervention *n* = 55 Waitlist control *n* = 55 Completion: 90 nurses (intervention *n* = 44; control *n* = 46)	Randomised control trial wait list control	8 week MBSR programme: adapted manualized Intervention consisted of eight 2-hour weekly group sessions; 20 minutes of formal mindfulness practice at home daily for 6 days/week for 8 weeks.	CD-RISC (25) revised to Chinese population **Other measures** Perceived Stress Scale (PSS) and Negative Affect Schedule (PANAS) McCloskey/Mueller Satisfaction Scale	T0 Baseline T1 Post intervention T2 3 months	The **IG** showed decreases in stress and negative affect and increases in positive affect and **resilience** after the intervention. 54.43 pre 57.98 post 59.73 mths follow up. No significant effect of group or time on resilience was identified between the two groups (*p* > .05), but the effect of the Group X Time interaction on resilience was statistically significant (*p* < .05). Significant differences in resilience were found between the two groups at the 3-month follow-up (*p* < .05). The levels of resilience were significantly different between T0 and T2 (*p* < .05). Not immediately after the intervention’ **Other outcomes IG** decreases in stress and negative effect post intervention
Magtibay et al. 2017 [53] USA	To assess the efficacy of blended learning to decrease stress and burnout among nurses using SMART	Transplant nurses and nurses in leadership roles (*n* = 50) complete T1 (*n* = 45) T2 (*n* = 40) T3 (*n* = 33)	Quantitative pre-test post test	CBT – SMART programme- condensed (12modules) and web based advised to complete in 8 weeks. 4 discussions with PI at weeks 8, 12, 16 and 20. 16 and 20 on phone.	CDRISC (2 item) **Other outcomes** Happiness, Stress, Anxiety Mindfulness and Burnout	8 weeks 12 sessions Telephone and in person follow up 8, 12, 16 and 20	Significant increases in scores at T2 and T3 but not immediately after the intervention Baseline mean = 6.2 Post intervention mean 6.3 Follow up mean = 6.7 (24 weeks) (*p* = 0.004)
Mealer et al. (2014) [54], USA	To determine if a multimodal resilience training program for ICU nurses was feasible to perform and acceptable to the study participants.	27 ICU nurses who were scored negative for being resilient (CDRISC 25 score 82 or less) randomized into intervention n= (14) and control (no intervention (*n* = 13)	Single centre Randomised control trial (no control)	12 week intervention comprising: Two-day workshop on resilience, psychological distress, self-care, mindfulness exercise and written exposure therapy. Post workshop: writing therapy (twelve 30-minute sessions), MBSR (15 min at least 3 times per week), 30 to 45 min of aerobic exercise (at least 3 days per week), and participated in an event-triggered cognitive behavioural therapy session.	CDRISC (25) **Other outcomes** Post Traumatic Diagnostic Scale(PDS), The Hospital and Anxiety Scale (HADS) The Maslach Burnout Inventory(MBI), The Client/Patient Satisfaction Questionnaire (CSQ 8)	Pre- intervention 1 week after intervention	Both the IG and CG had a significant improvement in resilience scores, but the change did not differ significantly between the IG and CG. Median score pre71 Post 78 *p* < 0.05 Other outcomes Significant reduction in PTSD symptoms in both groups IG group reduction in depression (*P* = .03) when compared to CG Informal qualitative review 2 days too long, need for booster sessions and benefits of group activity for mindfulness.
Mintz-Binder, Andersen et al. 2021 [55] USA	Investigating nurse resiliency utilizing a toolkit of stress-reducing interventions	148 nurses at 4 hospitals *n* = 77 completed the follow up survey	Quasi experimental pre-test post-test intervention pilot study	Provided Tool kits with written instructions for use over 6 weeks: included Lavender aromatherapy stick. Adult colouring book and pencils; recommendations for deep breathing; relaxation through guided meditation; Mind activity book; gaming apps.	CD-RISC (10) **Other outcomes** Likert scale measured stress levels (self) before and after the intervention	Baseline and post intervention. Participants reported when they used the toolkit at 10 time points over 6 weeks	Resiliency scores increased significantly after use at follow-up (df77 = −2.141, *P* < .02). Participants used breathing exercises more frequently (*P* = .031). Younger participants used the lavender inhaler for a progressively longer time as the study continued (*P* = .003). There was no statistical significance in resiliency scores related to non–work-related stressors or any other demographic variables at baseline df77 = − 2.141, *P* < .02)

## Results

A total of 3164 papers were identified. After the removal of duplicates 2919 were screened by title and 578 articles reviewed at abstract. Overall, 26 studies were included for full text review with 11 excluded with reasons. A total of 15 studies were included in the analysis (Fig. 1). 

### Study characteristics

Fifteen studies were included in this rapid review. Six studies were Randomised control trials [43, 45, 51, 52, 54, 50]. Four studies used Quasi Experimental designs [41, 46, 55, 42]. Five studies used a pre-test post-test survey design [44, 47–49, 53]. Most of the studies used convenience sampling with participants self-selected. The participants were mostly female ranging from 71 to 100%. The mean ages of participants when reported varied with mean age over 40 in three studies [47, 49, 50].

Over half of the studies took place in the USA (*n* = 8) [41, 44–46, 50–53]. Other countries represented were Iran (*n* = 1) [42], Germany (*n* = 1) [43], Australia (*n* = 2 [47, 49], United Kingdom [48], Korea [51] and China [52]. Overall, 1297 registered nurses participated in the studies. While all studies involved registered nurses, one study [44] also included four other healthcare professionals in a sample of 164 at one oncology centre. Therefore, the decision was made to include this study given the small number of non-nurses (4/164). Three studies looked specifically at nurses who were recently registered, two with a view to introducing the intervention as part of a pilot residency programme [41, 45, 46]. The general hospital was the setting for most of the studies with other settings including an oncology centre [44] and an academic medical centre [46, 55]. Within the hospital setting samples from specialist units were recruited such as specialist intensive or critical care units [54, 42], two mental health inpatient units [43, 49] and one unit with transplant nurses and those in leadership roles [53]. All studies aimed to determine the effectiveness of the interventions by changes in resiliency scores using a range of tools that have undergone psychometric testing for reliability and validity namely: the Connor Davidson Resilience Scale [9] (25 item, 10 item and 2 item), the Brief resilient Coping Scale [56], The Ego Resilience Scale [57] and the Workplace Resilience Inventory [58] with several studies using additional secondary outcome measures such as Stress, Compassion fatigue, Depression, Anxiety, Burnout, Satisfaction and Negative Affect.

### Demographics and attrition

In some studies where demographic data was reported analysis was undertaken primarily to describe the homogeneity between the populations in control and intervention groups [43, 52, 54]. There was no statistical difference found in the demographic data on resiliency scores in a quasi-experimental study (*n* = 328) [41]. However in one RCT (*n* = 196) pre intervention resiliency scores reported older nurses and those with more years in nursing to have higher resiliency scores [50].

Notable was the attrition in studies with larger number of participants [41, 45, 55, 50]. The highest attrition was 60% in a randomised control trial undertaken to determine the effectiveness of a three-hour resiliency community model training with nurses in two tertiary hospitals. Following randomisation only 40% of the sample attended the class or took part in one or more of the follow up surveys [50]. When comparing non attendees and those who attended there was no difference in age, years of experience or secondary traumatic stress scores. The non-attendees had slightly lower well-being and higher somatic stress scores. In a study evaluating the benefits of a tool kit to promote workplace resilience [55] the reasons for high attrition related to time of data collection coinciding with record levels of absenteeism from winter flu and overhauling of email systems. However, in a study replicating this attrition was still 57%, reasons suggested by the authors in discussion with nurse scholars, were that people may have joined to get the toolkit or professional development points [41]. An interesting finding from the pilot study was that when nurse scholars were on the hospital units promoting the resource the attrition level was less [55].

### The interventions

The content duration and the delivery of the interventions was variable across studies. Most of the interventions were delivered in groups (*n* = 13) with individual practice included and encouraged. Only two studies did not involve group activity, one which was a pilot for the larger study [41, 55]. In these two studies a tool kit of items to support resilience was given to participants following a video instruction. The tool kit included an adult colouring book, aromatherapy pen, breathing exercises, and links to various online resources. The participants recorded their use of the kit over 10 shifts using self-report measures relating to level of stress and frequency of use.

Most studies included an element of mindfulness and resiliency training, problem solving, breathing instruction, yoga, cognitive behavioural and interpersonal therapy. Manualised programmes such as Stress management and Resilience training (SMART), promoting adult resilience (PAR) and modifications such as the Mindfulness self-compassion programme (MSC) Mindfulness self-care (MSC) and mindfulness self-care and resiliency programme (MSCR) formed the basis of most interventions [45–48]. One mindfulness based intervention focused on resilience as a buffer to compassion fatigue [47]. Creative writing, adult colouring and aerobic exercise supplemented training programmes [41, 54, 55]. Some programmes offered links to relevant websites and compact discs of practices to boost resilience with a view to maintaining continuity through the intervention programme [48, 50]. A non-cognitive adaptation of mindfulness formed the basis of The Community Resilience Model (CRM) intervention which focused on developing sensory awareness techniques aiming to achieve emotional balance [50].

**Fig. 2:**
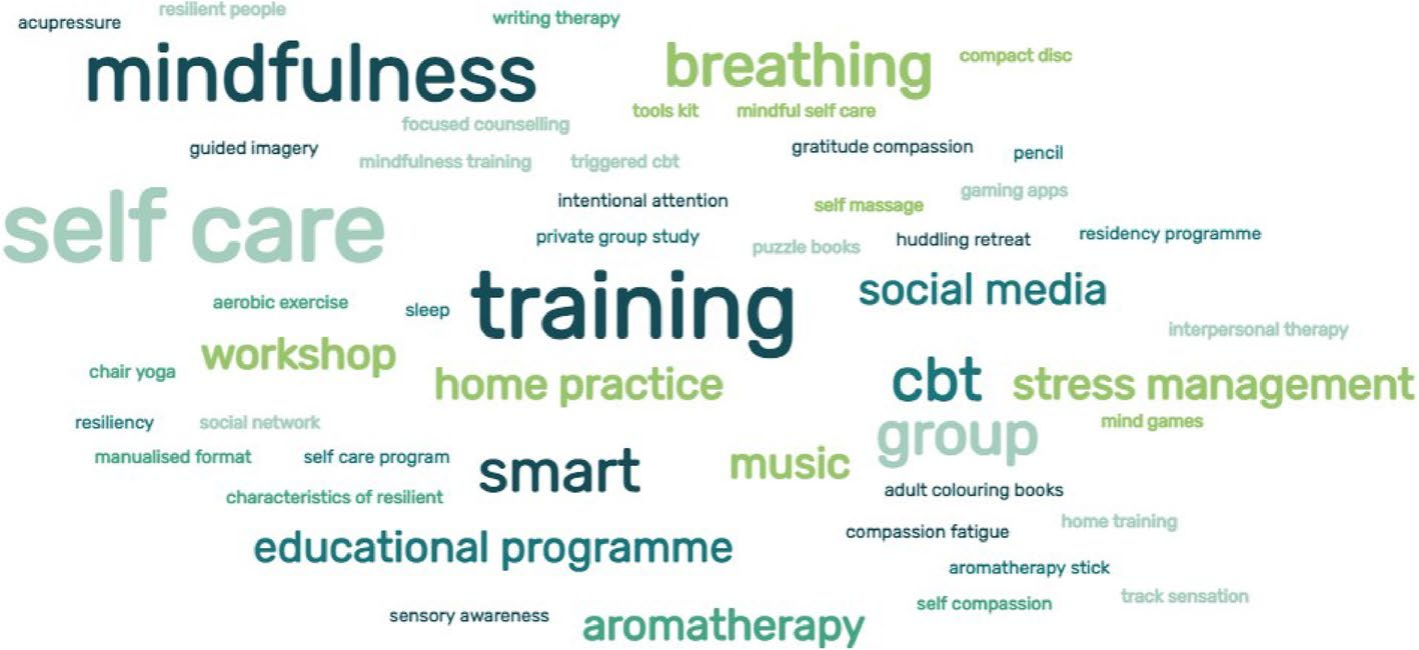
Word Cloud showing a range of interventions in reviewed studies

While face to face didactic teaching formed a significant part of most interventions a blended learning approach was used in one study [53]. In this study the SMART programme was adopted with the programme delivered in an online format and follow-up in person weekly sessions. To decrease attrition and respond to the busy lives of participants a 7 week manualised programme was adapted to a 2 day face to face programme 3 weeks apart for nurses in an acute mental health unit in Australia [49]. Social media was used for connection, group study and as a platform for hosting group activity in two studies [44, 51] The duration of the studies differed with the shortest being a 3 hour class with access to a mobile application relating to somatic responses to stress [50]. Seven studies were of an 8–12-week duration [43–45, 48, 51, 53, 54] with one study incorporating the SMART principles into residency programme monthly for 7 months [46]. Other studies duration were 4–6 weeks to include 10 shifts involving direct patient care [41, 55] and 12 hours over 4 weeks [47]. In one study, five teaching sessions each lasting 90–120 minutes covered aspects of resiliency, what makes a person resilient and how resilience may be supported through specific activities and practices [42]. Almost all studies incorporated some form of didactic instruction, many including individual self-practice and smaller group work. This was reinforced using mobile applications, instruction cards, books, handouts, and social networking. Social media was also used as a platform for smaller private group activity [44, 51]. Figure 2 presents a Word Cloud demonstrating the content and range of interventions outlined in the studies.

### Measures of resilience

The Connor Davidson resilience scale (CDRISC) was used in 12 out of the 15 studies, with six studies using the 25-item version [44, 45, 48, 52, 54, 42], five using the 10-item version [41, 46, 47, 55, 50] and one using the two-item version [53]. Of the six studies that used the CDRISC 25 item, five showed significant increases in resilience [44, 48, 52, 54, 42]. Despite an increase in resilience scores in Chesak’s pilot study, no statistical significance was demonstrated [45]. In a quasi-experimental study using the same intervention with novice nurses as part of a residency programme [46] attendance rates increased significantly as did the resilience levels in the intervention groups by comparison to the control group [46]. This was measured using the Connor Davidson 10 item Scale [59]. Four out of five studies measuring resilience with the Connor Davidson 10 item questionnaire reported significant increases in resiliency [41, 46, 55, 50]. The single study that demonstrated no significant changes in resiliency comprised a 12-hour mindfulness based training intervention, including a day workshop on compassion fatigue and weekly training sessions in mindfulness over 4 weeks [47]. The two item CDRISC scale [60] used to measure resilience demonstrated significant increases in resilience scores at T2 and T3 but not immediately after the intervention.

The brief resilient coping scale [56] was used to measure resilience levels at four time points following a 12 week training programme covering stress management, problem solving and solution focused counselling [43]. This demonstrated significant increases in resilience with effect sizes ranging from medium to large. Two further scales were used to evaluate the effects of interventions in the retrieved studies; the ego-resilience scale [57] which showed no significant differences following a 9 week intervention comprising a full day retreat and online or small group ‘huddling programme’ [51]. The Workplace Resilience Inventory (WRI) [58] was used to measure individual resilience factors and demonstrated a strong positive correlation between resilience factors and coping [49].

## Discussion

The results of this rapid review of the literature suggest that individual centred resilience interventions can improve nurse resilience. Thirteen of the fifteen studies selected demonstrated an increase in resilience scores as measured by validated and reliable scales demonstrating that the interventions worked in terms of increasing resilience. Previous reviews have highlighted the different interpretations of resilience that relate to protective and risk factors relating to mental health rather than the concept of resilience [13, 17] and the difficulties in determining what exactly is being measured. In this review the Connor Davidson Scale [9] was the primary scale used to measure resilience, this correlates with other reviews of resilience in health care professionals where the CD-RISC was the most commonly used measurement scale [7]. A methodological review of resilience scales found the CD-RISC to be in the top three for psychometric properties [61]. This scale measures resilience as encompassing personal attributes such as self-efficacy, and confidence but also recognises the importance of perception of experience and the importance of interconnectedness [9]. The scale focuses on strengths that enable people to not only recover but thrive in adversity. However as noted by Windle et al. [61] the scale relates to assets and resources, it may be considered to measure the process of resilience rather than resilience as an outcome. The innate nature of resilience makes objective outcome measurement difficult, therefore complementing the quantitative data with qualitative data could help discover what resilience as an outcome looks like and provide culturally and contextually relevant information that could inform the building of a resource for care home nurses.

While the intervention type, population and duration differed between the studies a central focus of the studies was developing self-awareness. Developing self-awareness enhanced control in stressful situations [54]. One of the most reproducible findings in resilience research is that the more control people have over stress situations the less negatively that stress will impact them [62]. Better understanding enabled such control in a study of nurses in a high acuity mental health setting where higher resilience resulted in positive coping following an educational intervention comprising cognitive behavioural and interpersonal therapy [49]. Awareness was developed through a tracking of sense in one study [50] recording of stress levels in another [41, 55] and training to understand stress [43, 45, 46, 42], and decrease compassion fatigue [47]. These individual level interventions aimed to make nurses aware of how stress impacts a person physically, mentally, and socially and how building resilience provides tools that can be transferred and accessed to help deal with adverse situations. As with other reviews of resilience in nursing and across health care demographic data is patchy and conflicting [6, 7]. As the determinants of resilience may be context specific [18] perhaps it is futile to compare across contexts, instead larger samples may provide more robust evidence as to how interventions can be tailored to demographic groups in similar contexts. Care homes nurses work in a unique environment where there is variation in the knowledge and skill set of those providing direct care of the resident [63], coupled with less health care support and high staff turnover [64, 65]. Being cognisant of these broader more structural variables that could impact not only the delivery of interventions but the ethos of the care home is vital in the design and delivery of targeted interventions.

All studies incorporated some element of mindfulness most focusing on a reframing of cognitive processes to focus on acceptance and being present. However, the medium for delivering the mindfulness practice differed and the level of cognitive reframing varied. In one study a large positive association between mindfulness and resilience (*r* = 0.66) was reported following an 8 week mindful self-compassion training course undertaken in the UK [48]. Mindfulness and resilience have been shown to conceptually overlap, with mindfulness focusing on cultivating positive emotions that help facilitate resilience [66]. The incorporation of mindfulness into a resiliency building programme could help in the reframing of thoughts by enabling.

’A reinterpretation of negative emotions as temporary visitors that will inevitably be replaced by other more welcome guests” [67].

In two studies the use of guided meditation, mind activity books and adult colouring books formed part of an individual intervention [41, 55]. A non-cognitive and novel operationalisation of mindfulness formed the basis of a single intervention lasting 3 h and demonstrating significant improvements in resilience [50]. While it is difficult to identify which of the interventions was most successful due to incomparable sample sizes, duration, and type of intervention, it appears that those interventions that focused on providing immediate ‘on the job’ fixes such as breathing, mindfulness and tracking exercises demonstrated higher scores especially when reviewed over time. Interestingly in one RCT with a multimodal intervention undertaken with nurses in ICU [54] both the control and the intervention group reported significant increases in resilience 1 week after the intervention. This could be explained by the group effect where the control group also benefited from the connectedness of their group. The authors also suggest some overlap with both interventions introducing mindful eating. Indeed, in a synthesis of systematic reviews on resilience in health professionals, feelings of support and connection embedded in workplace culture, significantly influenced individual resilience [68]. It would have been interesting to see if there was a difference between the intervention and control group some months later as this intervention incorporated both reactive (trigger focused CBT) and preventative (nutrition, sleep, exercise) elements. Indeed, in some of the studies that measured resilience at several time points, increases in resilience levels were noted after longer time frames rather than immediately after the intervention [52, 53]. This was also a finding in a RCT involving human service professionals where no instant increase in resilience was noted in the immediate aftermath of the intervention, but significant increases were noted at 4 months [69] suggesting that people who took part started to put into action the strategies they had learned through the intervention. Resilience in nursing viewed as a dynamic process [19] would suggest that time is required to develop skills that positively influence the appraisal of a stress situation and positive coping that results from that appraisal [70]. Indeed, the authors of a four-week intervention that showed no significant change in resiliency scores suggested that the brief duration of the intervention may not fully address the ‘character based ‘processes of resilience [47]. Therefore, longitudinal evaluation of interventions may demonstrate more positive results when participants can realise the benefit of newly acquired skills.

Adherence and engagement with the intervention varied across studies with high attrition levels in some interventions. Recommendations for further research suggest that future studies explore what level of engagement with an intervention is required to ensure a resilient outcome [46]. Informal feedback from one RCT revealed that training should be condensed to 1 day with more booster sessions that would help develop a professional network and enable group activity such as mindfulness [38]. The use of written pamphlets, social media, websites, mobile apps, and games with guides to meditation were used to support many of the interventions. These acted as a prompt to participate in studies, a means of debrief following intervention and as a reminder to self-practice. This is an important consideration in intervention development in promoting user engagement and within the care home setting visual aids in common spaces could act as a prompt to take time to be mindful or just to breathe. A large effect size was demonstrated in one of the included studies of newly qualified nurses where the intervention was incorporated into the residency programme and delivered over 7 months [46]. A programme delivered over a longer time span may address some of the concerns over the ability to influence the innate aspect of resilience with short interventions [7]. While none of the interventions were based totally online, the high attrition involved in many of the reviewed studies point to a need for alternate methods of intervention delivery. In a study of the acute effects of online mind-body skills training on resilience, mindfulness, and empathy (*n* = 513), the online training was seen to reach and positively affect the resilience levels of a diverse population and in particular those individuals who were stressed [71].

While interested in participating and getting immediate benefits, follow up engagement in some studies was low suggesting interventions should be flexible, readily accessible allowing engagement ‘where they are at’ [72]. Interestingly to address attrition Andersen [41] changed to a pen and paper record of resource use but this made no difference to attrition suggesting that the online recording was not an issue. The use of online and mobile technologies as a platform for delivering resilience interventions may be more cost effective and may reach a greater audience [73]. While this may be a way of minimising disruption in the care home environment where staffing levels are a constant challenge, some consideration needs to be given to the digital capability of the home and care home nurses. A negative correlation between age and informatics was reported in a review of issues affecting nurses ability to use digital technology [74]. In a recent review of care home nurses undertaken by the Queens Institute, 78% of nurses were aged over 45 yrs. [75], therefore interventions should be designed around available resources and may need to incorporate some learning on technologies. Reviews on the efficacy of interventions to foster resilience among health care professionals [17, 76] and other reviews specific to well-being in care home workers [33] suggest that co-designed adequately powered interventions will be more effective as the participants have local knowledge and can advocate for further resources if they are invested in the design and development of the resource.

### Strengths and limitations of the review

Limitations relate both to the review process and the individual studies extracted for review. Some of the reviewed studies used small sample sizes. As the influence of demographics on resilience in nursing is conflicting [6] adequate sample sizes with detailed demographics would provide more robust evidence of intervention effect and where interventions need to be tailored to support particular groups. The review is limited in not considering the most recent studies that are been developed or piloted, however the study time frame and funding did not allow for further searches.

As this was a rapid review of the literature and included reliable and validated scales a detailed quality assessment was not undertaken. The use of validated scales to measure resilience pre and post intervention primarily through quasi-experimental designs using valid and reliable tools adds rigour to the review. This provides best evidence as to the effect of individual interventions with the choice of the Connor Davidson Resilience scale in most studies addressing issues of varied interpretation of resilience. The scale authors are clear in their asset-based interpretation of resilience as the ability to positively adapt and potentially thrive in adversity [9]. While other reviews have noted findings as preliminary because of the use of pilot studies [7], two studies in this review were built on earlier pilot data with a resultant improvement in resiliency scores [41, 46]. The main improvements related to increasing the duration of the intervention and the measurement of longer-term outcomes.

## Conclusion

This review aimed to determine the effectiveness of interventions to promote resilience in registered nurses to inform the development of a resource to support the resilience of nurses working in care homes. The findings suggest that resilience can be enhanced through interventions. The high attrition in some larger studies suggests that intervention should be designed with the people who will use the resource, giving them something to take away or use ‘on site’ whenever it is required. Sensory awareness and multimodal interventions appeared to be more successful, perhaps appealing to different styles of learning and engagement. Integrating resilience training into programmes for new nurses might allow for the gradual development of resilience as a core capability. Care homes are heterogeneous in terms of staff, levels of care, location, and organisational structures [77], therefore, interventions aimed at supporting care home nurses need to be flexible and may benefit from co-design involving practitioners at a local level to champion the intervention [33, 76]. The results of this brief review show that resilience can be influenced positively through interventions, this knowledge can be used to inform the content of interventions in partnership with care home nurses who will know how best how they may be engaged in the unique context of the care home environment.

## Supplementary Information

**Additional file 1.** Search terms (Psych Info database).

**Additional file 2.** Brief review checklist.

## Data Availability

The supporting data are published in the body of the review and supplementary files.

## Abbreviations

CDRISC: Connor Davidson Resilience Scale
RCT: Randomised Control Trial
CBT: Cognitive Behavioural Therapy
ICU: Intensive Care Unit
U.K: United Kingdom
USA: United States of America
