# Supplementary material for: A rapid review of evaluated interventions to inform the development of a resource to support the resilience of care home nurses
Source: BMC Geriatr. 2023 May 5;23:275. doi: 10.1186/s12877-023-03860-y (PMC10162002; doi:10.1186/s12877-023-03860-y)
Supplement: Supplementary file 1 — Additional file 1. Search terms (Psych Info database). [file 12877_2023_3860_MOESM1_ESM.docx]

Additional File 1 Search terms (Psych Info database)

| **#** | **Searches** | **Results** |
| --- | --- | --- |
| 1 | nurse*.mp. or exp Nurses/ | 78606 |
| 2 | "health care professional*".mp. | 10129 |
| 3 | "health care worker*".mp. | 2531 |
| 4 | "care worker".mp. | 561 |
| 5 | exp Elder Care/ or “care home worker".mp. | 5279 |
| 6 | resilien*.mp. or exp "Resilience (Psychological)"/ | 39890 |
| 7 | exp Adaptation/ or adapt*.mp. | 252083 |
| 8 | exp Coping Behavior/ or "cope or coping".mp. | 50309 |
| 9 | hardiness.mp. or exp "Resilience (Psychological)"/ | 17583 |
| 10 | 6 or 7 or 8 or 9 | 317798 |
| 11 | exp Crisis Intervention/ or intervention*.mp. | 444357 |
| 12 | ("psychological intervention*" or "internet based intervention*").mp. [mp=title, abstract, heading word, table of contents, key concepts, original title, tests & measures, mesh] | 8014 |
| 13 | exp Initiative/ or initiative*.mp. | 46261 |
| 14 | evaluat*.mp. | 623911 |
| 15 | exp Nursing Homes/ or "nursing home staff".mp. | 9460 |
| 16 | exp Strategies/ or exp Coping Behavior/ or "strategy or strategies ".mp. | 104197 |
| 17 | exp "Resilience (Psychological)"/ or exp Personnel Training/ or "resilience training or resilience program*".mp. | 33985 |
| 18 | 11 or 12 or 17 | 472307 |
| 19 | 1 or 2 or 3 or 4 or 5 | 93889 |
| 20 | 15 or 19 | 101398 |
| 21 | 11 or 12 or 16 or 17 | 560866 |
| 22 | 10 and 14 and 20 and 21 | 679 |
| 23 | limit 22 to yr.="2010 -Current" | 430 |
