# Supplementary material for: A rapid review of evaluated interventions to inform the development of a resource to support the resilience of care home nurses
Source: BMC Geriatr. 2023 May 5;23:275. doi: 10.1186/s12877-023-03860-y (PMC10162002; doi:10.1186/s12877-023-03860-y)
Supplement: Supplementary file 2 — Additional file 2. Brief review checklist. [file 12877_2023_3860_MOESM2_ESM.docx]

Additional File 2 Brief Review Checklist

Abrami PC, Borokhovski E, Bernard RM, Wade CA, Tamim R, Persson T, et al. Issues in conducting and disseminating brief reviews of evidence. Evidence & policy: a journal of research, debate and practice. 2010;6(3):371-89.


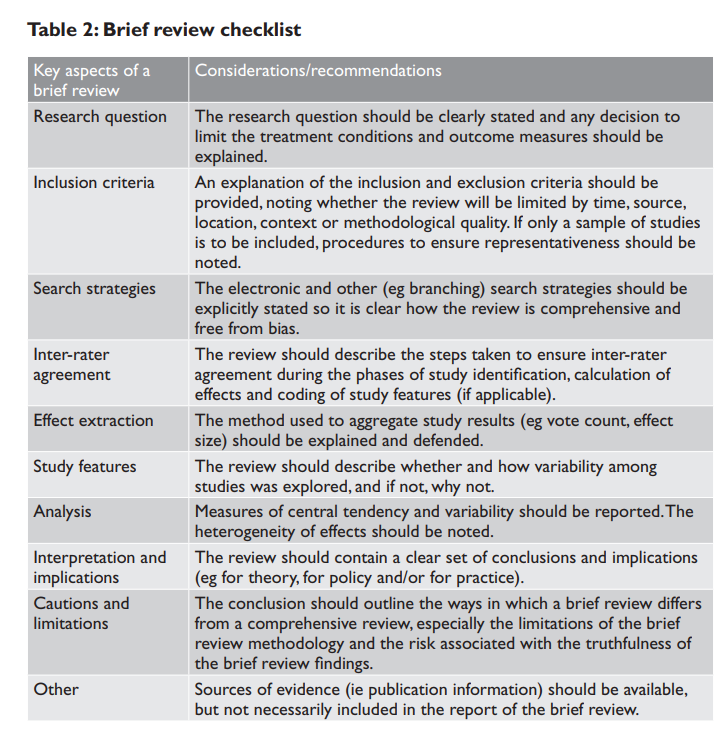


✓

✓

✓

✓

✓

n/a

n/a

✓

✓

✓

✓

✓
